# Supplementary material for: Seipin and the membrane-shaping protein Pex30 cooperate in organelle budding from the endoplasmic reticulum
Source: Nat Commun. 2018 Jul 27;9:2939. doi: 10.1038/s41467-018-05278-2 (PMC6063905; doi:10.1038/s41467-018-05278-2)
Supplement: Supplementary file 1 — Supplementary Information [file 41467_2018_5278_MOESM1_ESM.pdf]

Seipin and the membrane shaping protein Pex30 cooperate in organelle budding from the endoplasmic reticulum

Wang & Idrissi *et al*. Supplementary Information

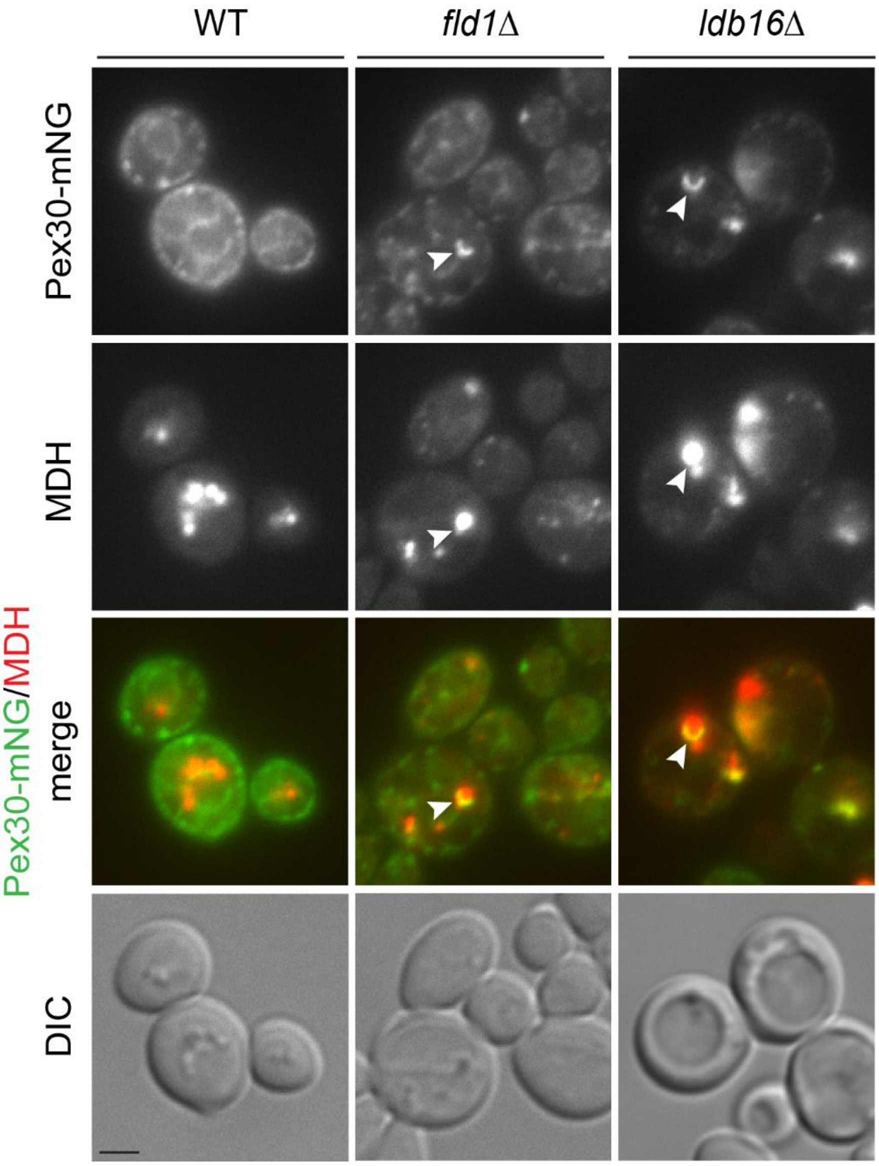

Supplementary Figure 1. Pex30 enrichment at ER-LD contacts (a) Pex30 is enriched at abnormal ER-LD contacts in *fld1*Δ and *ldb16*Δ mutants. Endogenous Pex30 was chromosomally tagged with monomeric Neon Green (Pex30-mNG). LDs were stained with the neutral lipid dye MDH. Bar 2μm.

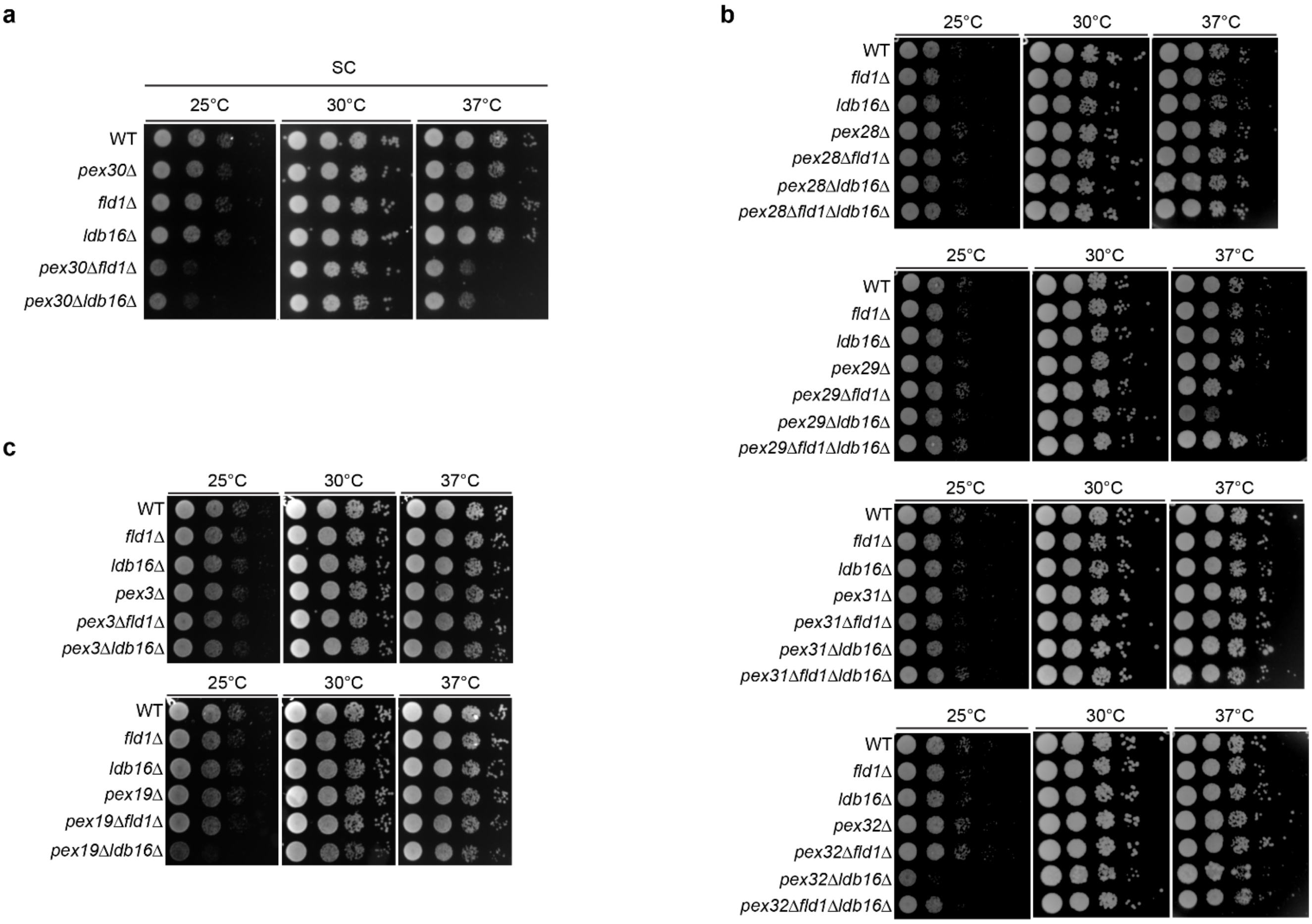

Supplementary Figure 2. Growth defect in cells lacking Pex30 and seipin is specific and not due to general loss of peroxisomal function. (a) 10-fold serial dilutions of cells with the indicated genotype were spotted on synthetic complete (SC) media and incubated at 25°C, 30°C or 37°C for 2 days. **(b)** Seipin does not interact genetically with other Pex30 family members. 10-fold serial dilutions of cells with the indicated genotype were spotted on YPD media and incubated at 25°C, 30°C or 37°C for 2 days. **(c)** Seipin does not interact genetically with components essential for peroxisome biogenesis. 10-fold serial dilutions of cells with the indicated genotype were spotted on YPD media and incubated at 25°C, 30°C or 37°C for 2 days.

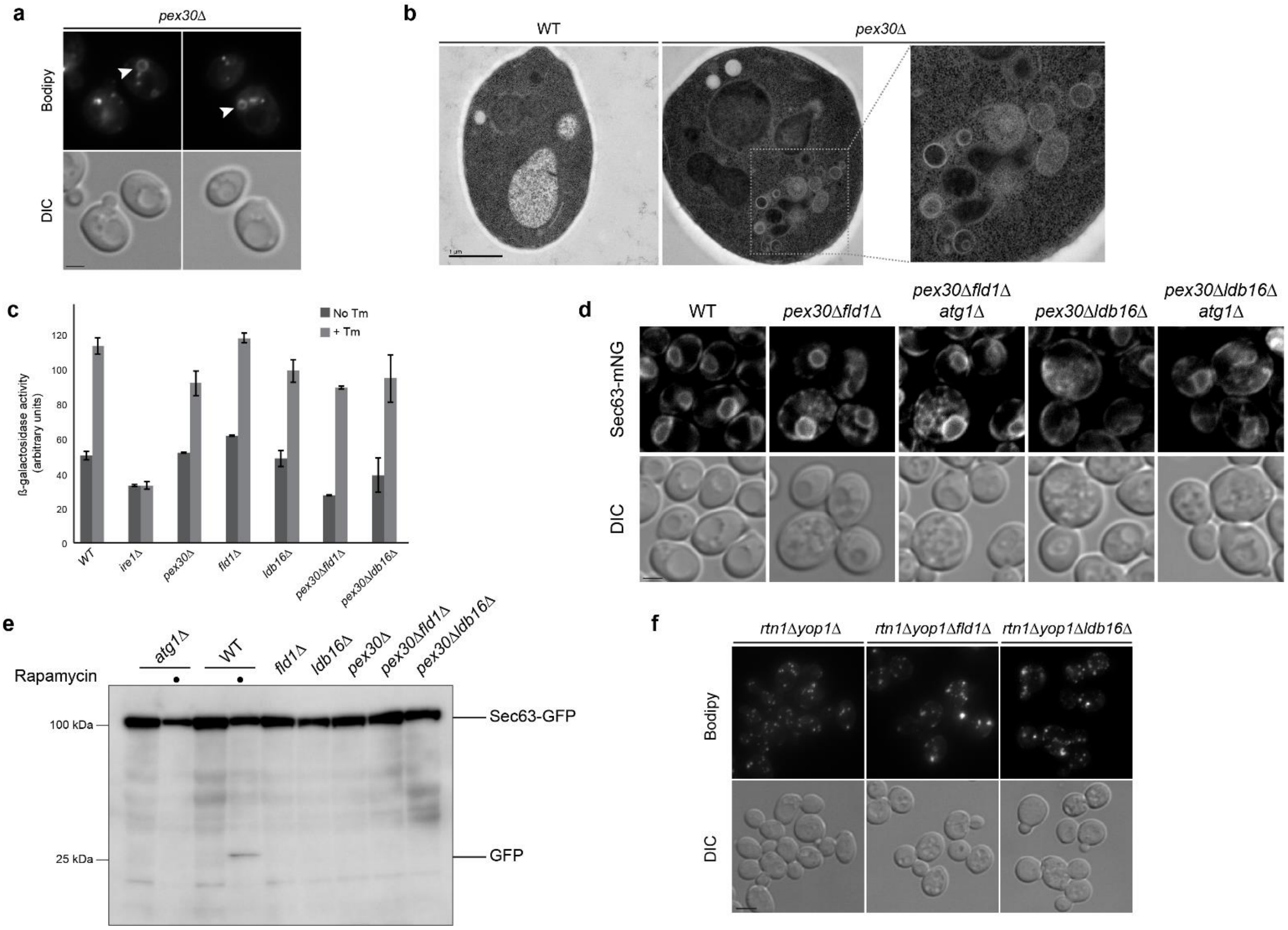

Supplementary Figure 3. (a) Although LDs appeared normal, a small fraction (4%) of *pex30*Δ cells displayed additional Bodipy-stained membranous-like structures (arrowheads). Bar 2μm. **(b)** Electron micrographs showing WT and *pex30*Δ cells. Please note that abnormal membranous structures were detected in a small fraction of *pex30*Δ cells. **(c)** *pex30Δfld1Δ* and *pex30Δldb16Δ* cells have basal UPR levels similar to WT and are capable of mounting an UPR response. UPR levels in cells with the indicated genotypes in absence (no tunicamycin, Tm) or presence (+ Tm) of ER stress, as determined by a ß-galactosidase reporter assay. The average of two experiments is shown; error bars represent standard deviation. **(d)** Analysis of ER morphology in cells of the indicated genotype. The ER marker protein, Sec63, was endogenously tagged with monomeric NeonGreen (Sec63-mNG). Bar, 2μm. **(e)** Sec63-GFP degradation was analysed in the indicated genotype by Western blotting, using an anti-GFP antibody. WT and *atg1Δ* cells were treated with 200ng/mL rapamycin for 12 h to induce autophagy. GFP: free GFP fragment generated by degradation of Sec63-GFP. **(f)** Mutations in the reticulons Rtn1 and Yop1, major ER shaping proteins, do not result in dispersed Bodipy structures. Analysis of LDs in cells with the indicated genotype after staining with the neutral lipid dye Bodipy493/503. Bar 2μm.

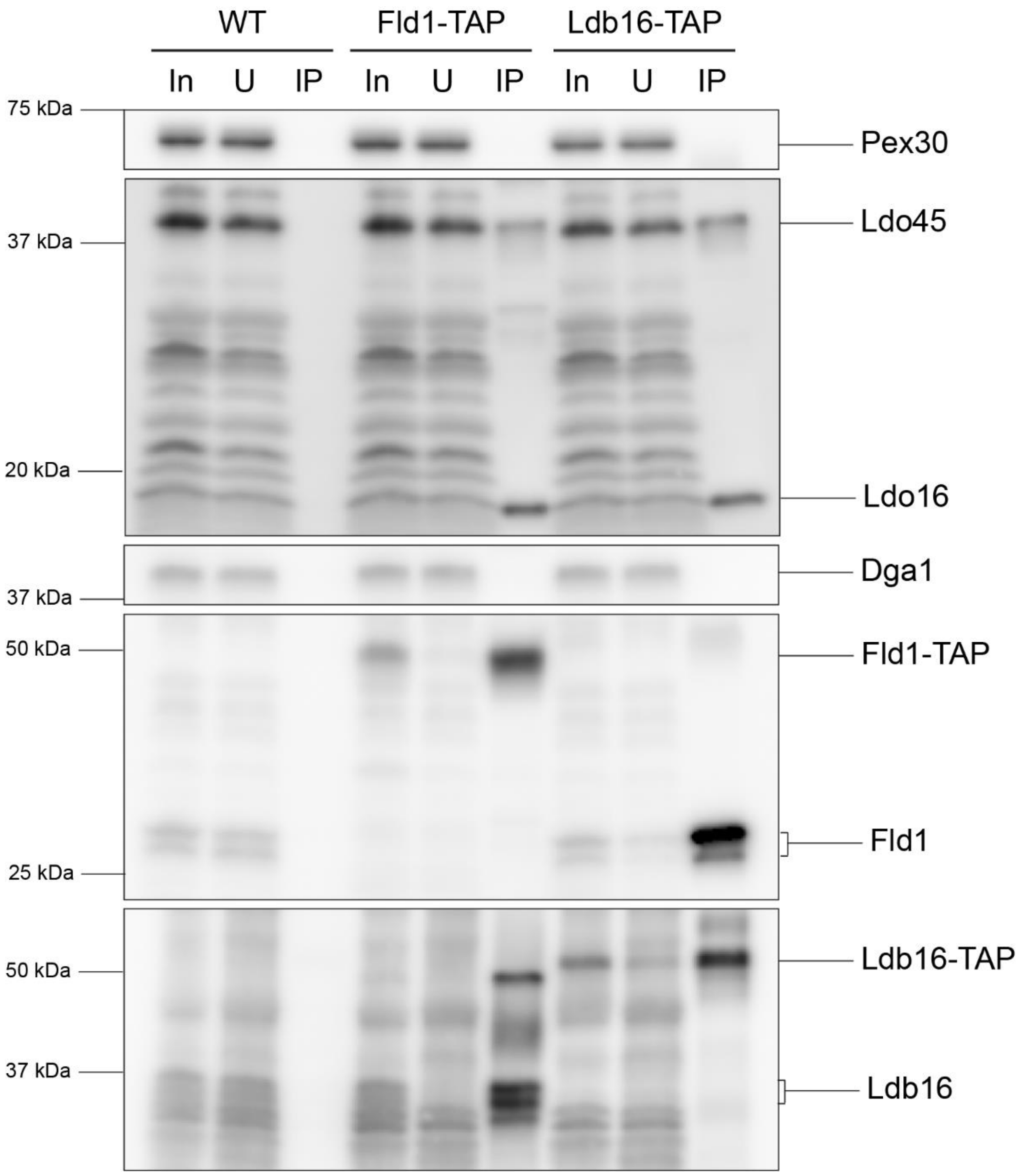

Supplementary Figure 4. Pex30 is not stably associated with the seipin complex. Detergent-solubilized extracts prepared from cells with the indicated genotype were immunoprecipitated (IP), and eluted proteins were analyzed by Western blotting. Endogenous Pex30 is not detected in seipin immunoprecipitates under conditions in which other components of the seipin complex components (Fld1, Ldb16, Ldo45 and Ldo16[56,70]) are detected.

a

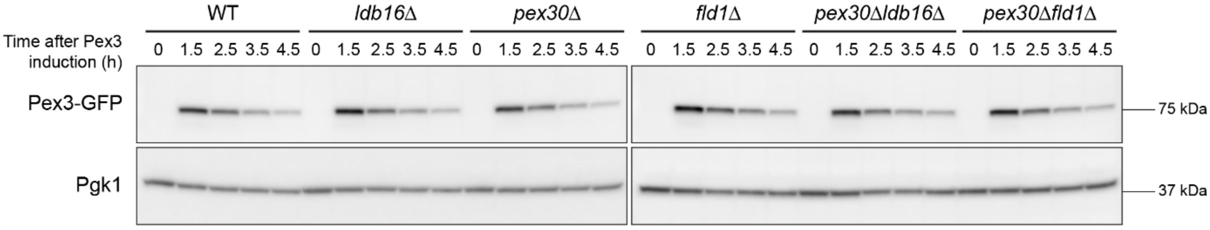

b

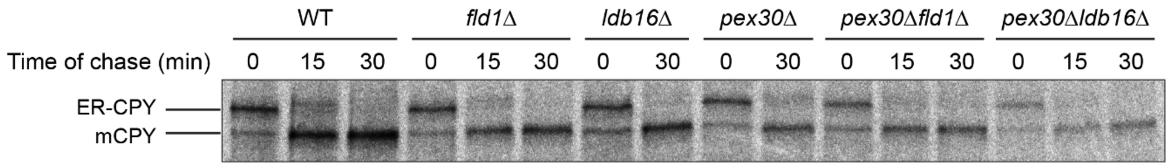

Supplementary Figure 5. (a) . Protein levels of GFP-Pex3 are similar in all cells during *de novo* peroxisome biogenesis. Levels of GFP-Pex3 at the indicated time points. Time point 0 corresponds to induction of GFP-Pex3 as depicted in Fig. 3a. At 30min time point GFP-Pex3 expression was repressed. Pgk1 was used as a loading control. **(b)** Maturation of endogenous Carboxypeptidase Y (CPY) in cells with the indicated genotype, as determined by pulse-chase. mCPY: mature, vacuolar form of CPY; ER-CPY: ER/golgi localized CPY.

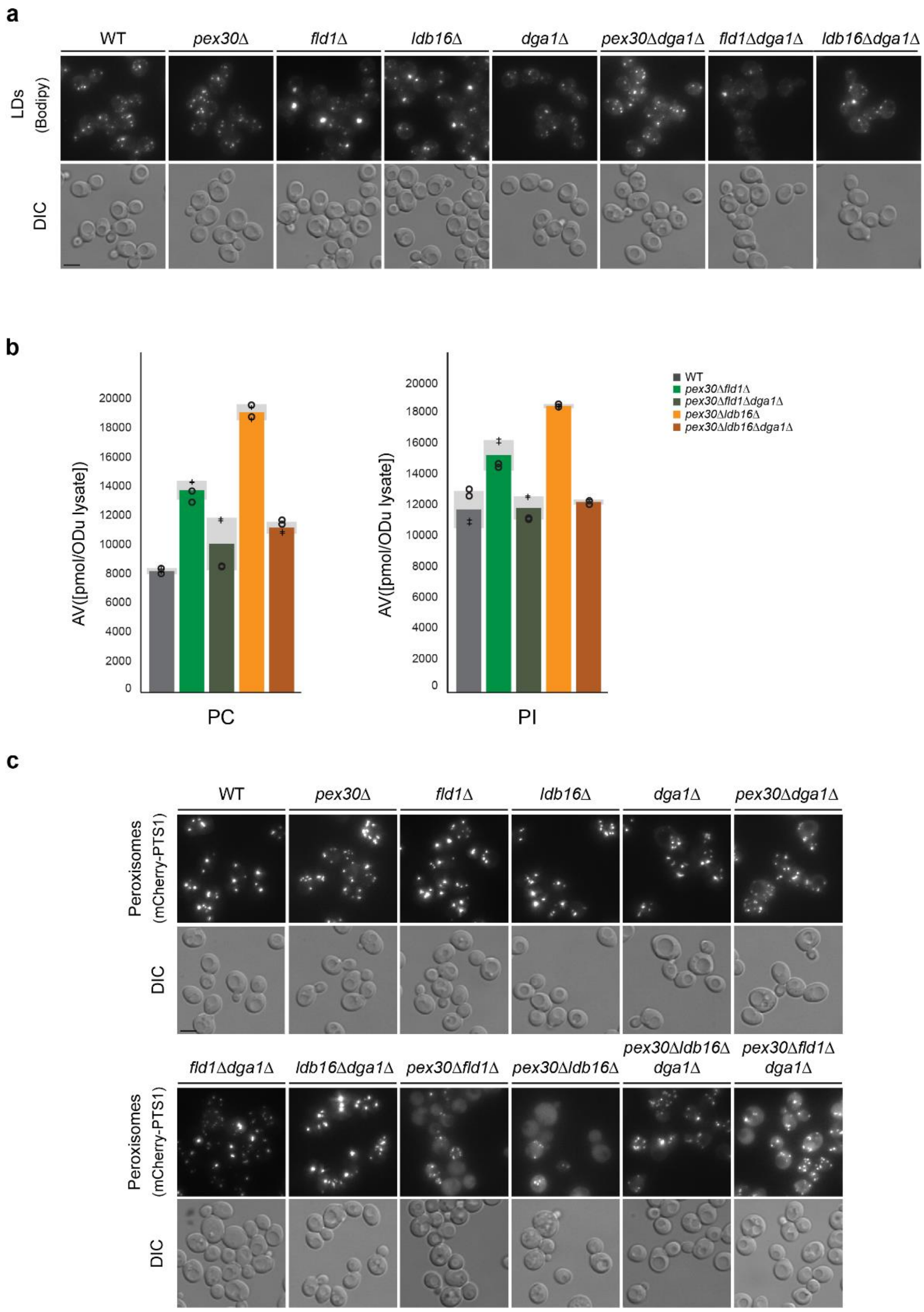

Supplementary Figure 6. (a) Cells with indicated genotype were stained with the neutral lipid dye Bodipy493/503. Bar 2 µm. **(b)** In the absence of seipin and Pex30, phospholipid levels are corrected by a reduction in TAG synthesis. Levels of the major phospholipids phosphotidylcholine (PC, left) and phosphatidylinositol (PI, right) in cells with the indicated genotype, as detected by mass spectrometry. Graphs correspond to the average of two biological and two technical repeats. The four individual measurements are displayed (+). Light grey bars indicate standard deviation. **(c)** Peroxisome biogenesis defect in cells lacking seipin and Pex30 is reversed by reducing TAG in the ER. Distribution of functional peroxisomes in cells with the indicated genotype analysed by steady-state localization of mCherry-PTS1. Bar 2µm.

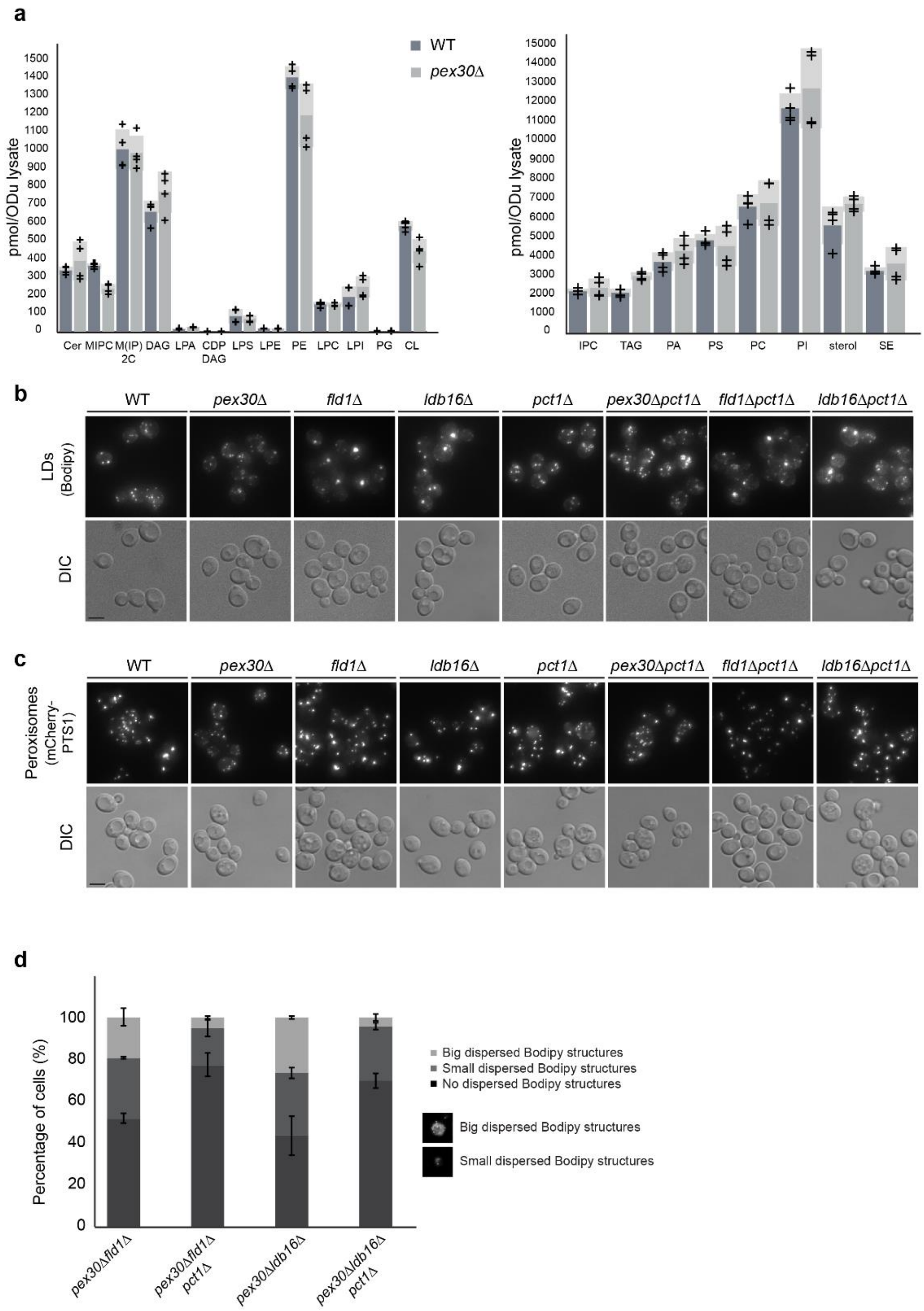

Supplementary Figure 7. (a) Global lipid composition of *pex30*Δ is similar to WT, as determined by shotgun lipidomics. Low and high abundance lipids are show on the left and right graphs, respectively. Graphs correspond to the average of two biological and two technical repeats. The four individual measurements are displayed (+). Light grey bars indicate standard deviation. **(b)** Analysis of LDs in cells with the indicated genotype after staining with the neutral lipid dye Bodipy493/503. Bar 2μm. **(c)** Distribution of functional peroxisomes in cells with the indicated genotype analysed by steady-state localization of mCherry-PTS1. Bar 2μm. **(d)** Quantification of dispersed Bodipy structures in cells with the indicated genotype. Data corresponds to the average of two experiments (>100cells/genotype/experiment were counted).

Supplementary Table 1- proteins increased in LDs isolated from *fld1Δ*, as determined by label free quantitative mass spectrometry.

Name		*fld1Δ*	
**Systematic**	**Standard**	**logFC**	**p Value**
YHL020C	OPI1	5.489566	1.46E-04
YLR324W	PEX30	5.063149	1.35E-05
YDR479C	PEX29	4.540304	8.46E-04
YNL044W	YIP3	4.432404	1.02E-05
YDR120C	TRM1	4.208153	9.99E-04
YML059C	NTE1	4.167676	3.16E-04
YPR097W	YPR097W	3.716108	1.17E-04
YLL040C	VPS13	2.827194	6.16E-04
YLR380W	CSR1	2.803219	4.00E-04
YGL140C	YGL140C	2.778662	4.69E-04
YNL287W	SEC21	2.568726	3.87E-04
YML020W	YML020W	2.546242	7.84E-04
YGR157W	CHO2	2.517351	4.84E-04
YGR202C	PCT1	2.473526	8.19E-04
YPR028W	YOP1	2.347473	8.20E-04
YDL029W	ARP2	2.114199	8.58E-04

Supplementary Table 2- proteins increased in LDs isolated from *ldb16Δ*, as determined by label free quantitative mass spectrometry.

Name		*ldb16Δ*	
**Systematic**	**Standard**	**logFC**	**p Value**
YHL020C	OPI1	4.20386	6.32E-06
YLR324W	PEX30	4.173158	4.11E-07
YML059C	NTE1	4.010077	9.84E-05
YNL044W	YIP3	3.824956	8.55E-07
YIL041W	GVP36	2.775928	8.10E-05
YPR097W	YPR097W	2.281816	6.38E-04
YGR202C	PCT1	2.180898	3.08E-04

A cut-off of LogFC ≥2 and p value < 0.001 was applied in both cases.

Supplementary Table 3. Yeast strains used in this study

Strains	Genotype
BY4741	*Mat a ura3Δ0his3Δ1leu2Δ0met15Δ0*
yPC3975	*Mat a ura3Δ0his3Δ1leu2Δ0met15Δ0fld1Δ::KAN*
yPC4246	*Mat a ura3Δ0his3Δ1leu2Δ0met15Δ0ldb16Δ::KAN*
yPC8808	*Mat a ura3Δ0his3Δ1leu2Δ0met15Δ0pex30Δ::KAN*
yPC8849	*Mat a ura3Δ0his3Δ1leu2Δ0met15Δ0pex30Δ::KAN fld1Δ::NAT*
yPC8851	*Mat α ura3Δ0his3Δ1leu2Δ0met15Δ0pex30Δ::KAN ldb16Δ::HYGB*
yPC8850	*Mat α ura3Δ0his3Δ1leu2Δ0met15Δ0pex30Δ::KAN fld1Δ::NAT ldb16Δ::HYGB*
yPC5561	*Mat a ura3Δ0his3Δ1leu2Δ0met15Δ0* PEX30-GFP-HIS3
yPC5680	*Mat ? ura3Δ0his3Δ1leu2Δ0met15Δ0fld1Δ::NAT* PEX30-GFP-HIS3
yPC5678	*Mat ? ura3Δ0his3Δ1leu2Δ0met15Δ0ldb16Δ::HYGB* PEX30-GFP-HIS3
yPC10657	*Mat a ura3Δ0his3Δ1leu2Δ0met15Δ0* PEX30-mNeonGreen-HIS3
yPC10676	*Mat ? ura3Δ0his3Δ1leu2Δ0met15Δ0fld1Δ::NAT* PEX30-mNeonGreen-HIS3
yPC10678	*Mat ? ura3Δ0his3Δ1leu2Δ0met15Δ0ldb16Δ::HYGB* PEX30-mNeonGreen-HIS3
yPC9035	*Mat a ura3Δ0his3Δ1leu2Δ0met15Δ0pex3Δ::KAN*
yPC9880	*Mat ? ura3Δ0his3Δ1leu2Δ0met15Δ0pex3Δ::KANfld1Δ::NAT*
yPC9881	*Mat ? ura3Δ0his3Δ1leu2Δ0met15Δ0pex3Δ::KANldb16Δ::HYGB*
yPC9838	*Mat a ura3Δ0his3Δ1leu2Δ0met15Δ0pex19Δ::KAN*
yPC9858	*Mat ? ura3Δ0his3Δ1leu2Δ0met15Δ0pex19Δ::KANfld1Δ::NAT*
yPC9860	*Mat ? ura3Δ0his3Δ1leu2Δ0met15Δ0pex19Δ::KANldb16Δ::HYGB*
yPC10902	*Mat a ura3Δ0his3Δ1leu2Δ0met15Δ0NAT*::Gal1::GFP-Pex3 mCherry-PTS1::HIS3
yPC10904	*Mat a ura3Δ0his3Δ1leu2Δ0met15Δ0ldb16Δ::HYGB NAT*::Gal1::GFP-Pex3 mCherry-PTS1::HIS3
yPC10906	*Mat ? ura3Δ0his3Δ1leu2Δ0met15Δ0pex30Δ::KAN NAT*::Gal1::GFP-Pex3 mCherry-PTS1::HIS3
yPC10909	*Mat ? ura3Δ0his3Δ1leu2Δ0met15Δ0pex30Δ::KAN ldb16Δ::HYGB NAT*::Gal1::GFP-Pex3 mCherry-PTS1::HIS3
yPC10964	*Mat ? ura3Δ0his3Δ1leu2Δ0met15Δ0pex30Δ::KANfld1Δ::NAT NAT*::Gal1::GFP-Pex3 mCherry-PTS1::HIS3
yPC10965	*Mat ? ura3Δ0his3Δ1leu2Δ0met15Δ0fld1Δ::NAT NAT*::Gal1::GFP-Pex3 mCherry-PTS1::HIS3
yPC10613	*Mat a ura3Δ0his3Δ1leu2Δ0met15Δ0* SEC63-mNeonGreen-HIS3
yPC10614	*Mat a ura3Δ0his3Δ1leu2Δ0met15Δ0pex30Δ::KAN* SEC63-mNeonGreen-HIS3
yPC10615	*Mat a ura3Δ0his3Δ1leu2Δ0met15Δ0fld1Δ::KAN* SEC63-mNeonGreen-HIS3
yPC10616	*Mat a ura3Δ0his3Δ1leu2Δ0met15Δ0ldb16Δ::KAN* SEC63-mNeonGreen-HIS3
yPC10617	*Mat a ura3Δ0his3Δ1leu2Δ0met15Δ0pex30Δ::KAN fld1Δ::NAT* SEC63-mNeonGreen-HIS3
yPC10618	*Mat α ura3Δ0his3Δ1leu2Δ0met15Δ0pex30Δ::KAN ldb16Δ::HYGB* SEC63-mNeonGreen-HIS3
yPC10692	*Mat ? ura3Δ0his3Δ1leu2Δ0met15Δ0* FLD1-tdTomato-HIS3 *NAT*::Gal1::GFP-Pex3
yPC4060	*Mat a ura3Δ0his3Δ1leu2Δ0met15Δ0dga1Δ::KAN*
yPC5416	*Mat ? ura3Δ0his3Δ1leu2Δ0met15Δ0dga1Δ::NAT ldb16Δ::HYGB*
yPC10625	*Mat ? ura3Δ0his3Δ1leu2Δ0met15Δ0pex30Δ::KAN dga1Δ::KAN*
yPC10626	*Mat ? ura3Δ0his3Δ1leu2Δ0met15Δ0pex30Δ::KAN dga1Δ::KANldb16Δ::HYGB*
yPC10695	*Mat ? ura3Δ0his3Δ1leu2Δ0met15Δ0dga1Δ::NAT fld1Δ::HYGB*
yPC10694	*Mat ? ura3Δ0his3Δ1leu2Δ0met15Δ0 pex30Δ::KAN dga1Δ::NAT fld1Δ::HYGB*
yPC10718	*Mat ? ura3Δ0his3Δ1leu2Δ0met15Δ0* mCherry-PTS1-HIS3
yPC10719	*Mat ? ura3Δ0his3Δ1leu2Δ0met15Δ0 pex30Δ::KAN* mCherry-PTS1-HIS3
yPC10720	*Mat ? ura3Δ0his3Δ1leu2Δ0met15Δ0 ldb16Δ::HYGB* mCherry-PTS1-HIS3
yPC10721	*Mat ? ura3Δ0his3Δ1leu2Δ0met15Δ0 dga1Δ::NAT* mCherry-PTS1-HIS3
yPC10723	*Mat ? ura3Δ0his3Δ1leu2Δ0met15Δ0 ldb16Δ::HYGB dga1Δ::NAT* mCherry-PTS1-HIS3
yPC10724	*Mat ? ura3Δ0his3Δ1leu2Δ0met15Δ0 pex30Δ::KAN dga1Δ::NAT* mCherry-PTS1-HIS3
yPC10725	*Mat ? ura3Δ0his3Δ1leu2Δ0met15Δ0 pex30Δ::KAN ldb16Δ::HYGB* mCherry-PTS1-HIS3
yPC10726	*Mat ? ura3Δ0his3Δ1leu2Δ0met15Δ0 pex30Δ::KAN ldb16Δ::HYGB dga1Δ::NAT* mCherry-PTS1-HIS3
yPC5251	*Mat a ura3Δ0his3Δ1leu2Δ0met15Δ0pct1Δ::KAN*
yPC5631	*Mat ? ura3Δ0his3Δ1leu2Δ0met15Δ0pct1Δ::KAN fld1Δ::NAT*
yPC10622	*Mat α ura3Δ0his3Δ1leu2Δ0met15Δ0pct1Δ::KAN pex30Δ::HIS3*

yPC10623	*Mat ? ura3Δ0his3Δ1leu2Δ0met15Δ0pct1Δ::KAN pex30Δ::HIS3 fld1Δ::NAT*
yPC10700	*Mat ? ura3Δ0his3Δ1leu2Δ0met15Δ0 pex30Δ::HIS3pct1Δ::KAN ldb16Δ::HYGB*
yPC10702	*Mat ? ura3Δ0his3Δ1leu2Δ0met15Δ0 pct1Δ::KAN ldb16Δ::HYGB*
yPC10716	*Mat ? ura3Δ0his3Δ1leu2Δ0met15Δ0 pct1Δ::KAN* mCherry-PTS1-HIS3
yPC10713	*Mat ? ura3Δ0his3Δ1leu2Δ0met15Δ0 pct1Δ::KAN ldb16Δ::HYGB* mCherry-PTS1-HIS3
yPC10714	*Mat ? ura3Δ0his3Δ1leu2Δ0met15Δ0 pex30Δ::HIS3pct1Δ::KAN* mCherry-PTS1-HIS3
yPC10711	*Mat ? ura3Δ0his3Δ1leu2Δ0met15Δ0 pex30Δ::HIS3 ldb16Δ::HYGB pct1Δ::KAN* mCherry-PTS1-HIS3
yPC10963	*Mat ? ura3Δ0his3Δ1leu2Δ0met15Δ0 fld1Δ::NAT* mCherry-PTS1-HIS3
yPC10746	*Mat ? ura3Δ0his3Δ1leu2Δ0met15Δ0 pex30Δ::HIS3fld1Δ::NAT* mCherry-PTS1-HIS3
yPC10748	*Mat ? ura3Δ0his3Δ1leu2Δ0met15Δ0 fld1Δ::HYGB dga1Δ::NAT* mCherry-PTS1-HIS3
yPC10744	*Mat ? ura3Δ0his3Δ1leu2Δ0met15Δ0 pct1Δ::KAN fld1Δ::NAT* mCherry-PTS1-HIS3
yPC10750	*Mat ? ura3Δ0his3Δ1leu2Δ0met15Δ0 pex30Δ::KANfld1Δ::HYGB dga1Δ::NAT* mCherry-PTS1-HIS3
yPC10745	*Mat ? ura3Δ0his3Δ1leu2Δ0met15Δ0 pex30Δ::HIS3pct1Δ::KAN fld1Δ::NAT* mCherry-PTS1-HIS3
yPC10688	*Mat a ura3Δ0his3Δ1leu2Δ0met15Δ0* GFP-PEX14-URA
yPC10689	*Mat ? ura3Δ0his3Δ1leu2Δ0met15Δ0 fld1Δ::NAT* GFP-PEX14-URA
yPC10690	*Mat ? ura3Δ0his3Δ1leu2Δ0met15Δ0pex30Δ::KAN fld1Δ::NAT* GFP-PEX14-URA
yPC10762	*Mat a ura3Δ0his3Δ1leu2Δ0met15Δ0pex30Δ::KAN* GFP-PEX14-URA
yPC10974	*Mat a ura3Δ0his3Δ1leu2Δ0met15Δ0ldb16Δ::KAN* GFP-PEX14-URA
yPC11048	*Mat a ura3Δ0his3Δ1leu2Δ0met15Δ0pex30Δ::KAN ldb16::HYGB* GFP-PEX14-URA
yPC10893	*Mat ? ura3Δ0his3Δ1leu2Δ0met15Δ0 fld1::HYGB dga1::NAT* Sec63-mNeonGreen-HIS
yPC10894	*Mat ? ura3Δ0his3Δ1leu2Δ0met15Δ0 pex30::KAN fld1::HYGB dga1::NAT* Sec63-mNeonGreen-HIS
yPC10660	*Mat a ura3Δ0his3Δ1leu2Δ0met15Δ0 dga1::KAN* Sec63-mNeonGreen-HIS
yPC10661	*Mat ? ura3Δ0his3Δ1leu2Δ0met15Δ0 dga1::NAT ldb16::HYGB* Sec63-mNeonGreen-HIS
yPC10662	*Mat ? ura3Δ0his3Δ1leu2Δ0met15Δ0 pex30::KAN dga1::NAT* Sec63-mNeonGreen-HIS
yPC10663	*Mat ? ura3Δ0his3Δ1leu2Δ0met15Δ0 pex30::KAN dga1::NAT ldb16::HYGB* Sec63-mNeonGreen-HIS
yPC10895	*Mat a ura3Δ0his3Δ1leu2Δ0met15Δ0* PEX30-mNeonGreen-HIS SEC63-mCherry-URA
yPC10896	*Mat ? ura3Δ0his3Δ1leu2Δ0met15Δ0 fld1::NAT* PEX30-mNeonGreen-HIS SEC63-mCherry-URA
yPC10897	*Mat ? ura3Δ0his3Δ1leu2Δ0met15Δ0 ldb16::HYGB* PEX30-mNeonGreen-HIS SEC63-mCherry-URA
yPC3421	*Mat a ura3Δ0his3Δ1leu2Δ0met15Δ0* LDB16-TAP-HIS5
yPC3389	*Mat a ura3Δ0his3Δ1leu2Δ0met15Δ0* FLD1-TAP-HIS5
yPC10698	*Mat a ura3Δ0his3Δ1leu2Δ0met15Δ0* PEX30-mNeonGreen-HIS FLD1-tdTomato-HIS
yPC1573	*Mat a ura3Δ0his3Δ1leu2Δ0met15Δ0* Sec63-GFP-HIS5
yPC8122	*Mat a ura3Δ0his3Δ1leu2Δ0met15Δ0 fld1::NAT* Sec63-GFP-HIS5
yPC8121	*Mat a ura3Δ0his3Δ1leu2Δ0met15Δ0 ldb16::NAT* Sec63-GFP-HIS5
yPC11050	*Mat a ura3Δ0his3Δ1leu2Δ0met15Δ0 pex30::KAN* Sec63-GFP-HIS5
yPC11051	*Mat a ura3Δ0his3Δ1leu2Δ0met15Δ0 pex30::KAN fld1::NAT* Sec63-GFP-HIS5
yPC11053	*Mat a ura3Δ0his3Δ1leu2Δ0met15Δ0 pex30::KAN ldb16::NAT* Sec63-GFP-HIS5
yPC11087	*Mat a ura3Δ0his3Δ1leu2Δ0met15Δ0 atg1::KAN* Sec63-GFP-HIS5
yPC11080	*Mat ? ura3Δ0his3Δ1leu2Δ0met15Δ0 pex30::KAN* PEX14-GFP-URA SEC63-tdTomato-HIS
yPC11081	*Mat ? ura3Δ0his3Δ1leu2Δ0met15Δ0* PEX14-GFP-URA SEC63-tdTomato-HIS
yPC11082	*Mat ? ura3Δ0his3Δ1leu2Δ0met15Δ0 ldb16::KAN* PEX14-GFP-URA SEC63-tdTomato-HIS
yPC11083	*Mat a ura3Δ0his3Δ1leu2Δ0met15Δ0pex30Δ::KAN atg1::HYGB* SEC63-mNeonGreen-HIS3
yPC11084	*Mat a ura3Δ0his3Δ1leu2Δ0met15Δ0fld1Δ::KAN atg1::HYGB* SEC63-mNeonGreen-HIS3
yPC11085	*Mat a ura3Δ0his3Δ1leu2Δ0met15Δ0ldb16Δ::KAN atg1::HYGB* SEC63-mNeonGreen-HIS3
yPC11086	*Mat α ura3Δ0his3Δ1leu2Δ0met15Δ0pex30Δ::KAN ldb16Δ::HYGB atg1::NAT* SEC63-mNeonGreen-HIS3
yPC11088	*Mat a ura3Δ0his3Δ1leu2Δ0met15Δ0pex30Δ::KAN fld1Δ::NAT atg1::HYGB* SEC63-mNeonGreen-HIS3
yPC11089	*Mat ? ura3Δ0his3Δ1leu2Δ0met15Δ0 pex30::KAN fld1::NAT* PEX14-GFP-URA SEC63-tdTomato-HIS
yPC11090	*Mat ? ura3Δ0his3Δ1leu2Δ0met15Δ0 fld1::NAT* PEX14-GFP-URA SEC63-tdTomato-HIS
yPC8806	*Mat a ura3Δ0his3Δ1leu2Δ0met15Δ0pex28Δ::KAN*

yPC8845	*Mat ? ura3Δ0his3Δ1leu2Δ0met15Δ0pex28Δ::KAN fld1::NAT*
yPC8844	*Mat ? ura3Δ0his3Δ1leu2Δ0met15Δ0pex28Δ::KAN ldb16::HYGB*
yPC8843	*Mat ? ura3Δ0his3Δ1leu2Δ0met15Δ0pex28Δ::KAN fld1::NAT ldb16::HYGB*
yPC8807	*Mat a ura3Δ0his3Δ1leu2Δ0met15Δ0pex29Δ::KAN*
yPC8848	*Mat ? ura3Δ0his3Δ1leu2Δ0met15Δ0pex29Δ::KAN fld1::NAT*
yPC8847	*Mat ? ura3Δ0his3Δ1leu2Δ0met15Δ0pex29Δ::KAN ldb16::HYGB*
yPC8846	*Mat ? ura3Δ0his3Δ1leu2Δ0met15Δ0pex29Δ::KAN fld1::NAT ldb16::HYGB*
yPC8809	*Mat a ura3Δ0his3Δ1leu2Δ0met15Δ0pex31Δ::KAN*
yPC8855	*Mat ? ura3Δ0his3Δ1leu2Δ0met15Δ0pex31Δ::KAN fld1::NAT*
yPC8854	*Mat ? ura3Δ0his3Δ1leu2Δ0met15Δ0pex31Δ::KAN ldb16::HYGB*
yPC8856	*Mat ? ura3Δ0his3Δ1leu2Δ0met15Δ0pex31Δ::KAN fld1::NAT ldb16::HYGB*
yPC8810	*Mat a ura3Δ0his3Δ1leu2Δ0met15Δ0pex32Δ::KAN*
yPC8858	*Mat ? ura3Δ0his3Δ1leu2Δ0met15Δ0pex32Δ::KAN fld1::NAT*
yPC8860	*Mat ? ura3Δ0his3Δ1leu2Δ0met15Δ0pex32Δ::KAN ldb16::HYGB*
yPC8857	*Mat ? ura3Δ0his3Δ1leu2Δ0met15Δ0pex32Δ::KAN fld1::NAT ldb16::HYGB*
